# The complete mitochondrial genome of the burrowing ghost shrimp, *Nihonotrypaea harmandi* (Bouvier, 1901), (Crustacea, Decapoda, Axiidea, Callianassidae) – a validation of the genus and species classifications

**DOI:** 10.1080/23802359.2017.1318676

**Published:** 2017-04-24

**Authors:** Akinori Yamada, Rei Somiya, Natsuki Ikeda, Akio Tamaki

**Affiliations:** Graduate School of Fisheries and Environmental Sciences, Nagasaki University, Nagasaki, Japan

**Keywords:** *Nihonotrypaea*, genome, mitochondria, Callianassidae, ghost shrimp

## Abstract

The complete mitochondrial genome of the burrowing ghost shrimp *Nihonotrypaea harmandi* was reconstructed using the Illumina HiSeq platform. The genome was 15,272 bp in length made up of 37 mitochondrial genes (13 CDSs, 22 tRNAs, and 2 rRNAs) in the same order as other *Nihonotrypaea* species. Phylogenetic analyses suggested that *Nihonotrypaea* is a valid genus, and that *N. harmandi* can be phylogenetically marginally separated from *N. japonica*, though some authors considered the former as a synonym of the latter.

The ghost and mud shrimps (former infraorder Thalassinidea, *cf*. Poore et al. 2014) play an important role as ecosystem engineers and affect ecosystem processes and community structure (Pillay & Branch 2011). The genus *Nihonotrypaea* (Manning & Tamaki 1998) is a callianassid ghost shrimp, and distinguished from other genera of the same subfamily, Callianassinae, by the appendices internae projecting from the margin of the endopod of pleopods 3–5 (Manning & Tamaki 1998). The status of *Nihonotrypaea* is, however, controversial, being regarded as a synonym of *Trypaea* Dana, 1852 (Sakai 2011). Currently, six species of *Nihonotrypaea* are described from the Northwest Pacific, of which three species, *N. harmandi* (Bouvier 1901), *N. japonica* (Ortmann 1891), *N. petalura* (Stimpson 1860), are distributed in an estuarine system in mid-western Kyushu, southern Japan, each inhabiting different environmental conditions (Tamaki et al. 1999), whereas Sakai (2011) considered *N. harmandi* and *N. japonica* belonging to one single species, *Nihonotrypaea* (= *Trypaea*) *japonica.* Here we determined the complete mitochondrial genome of *N. harmandi*, which was phylogenetically analyzed in order to clarify the status of the genus *Nihonotrypaea* and the relationships between *N. harmandi* and *N. japonica*.

An approximate of 30 mg of a male’s major cheliped-muscle was dissected from a live specimen of *N. harmandi* (Specimen Voucher: Nagasaki University #Call160314) collected from an intertidal sandflat in Koyagi, Nagasaki (129°47.4′E, 32°41.4′N) on 22 January 2016. Total DNA was extracted, and whole genome sequencing was outsourced to Macrogen (Seoul, South Korea). A total of 65M 101-bp paired-end reads generated by Illumina HiSeq 4000 were assembled using IDBA_UD (Peng et al. 2012). A circular contig which agreed with the known *Nihonotrypaea* mitochondrial genomes was annotated with MITOS (Bernt et al. 2012) followed by manual validation of the coding regions using the reference genomes. Phylogenetic analyses were conducted using MEGA7 (Kumar et al. 2016).

The complete mitochondrial genome of *N. harmandi* was 15,272 bp in length (GenBank accession number: LC221567), and contained 37 mitochondrial genes (13 CDSs, 22 tRNAs, and 2 rRNAs) in the same order as *N. japonica* (accession number: KC236422) and *N. thermophile* (accession number: JN897380). A phylogenetic tree reconstructed from a combined analysis of 13 CDSs and 2 rRNAs agreed with the classification of genera by WoRMS Editorial Board (2017) (Figure 1). Furthermore, despite the considerable morphological similarities between the species of *Nihonotrypaea* and *Biffarius* (*cf.* Liu & Liu 2014), the two genera formed distinct monophyletic clades that are well-separated from each other, suggesting the validity of the genus *Nihonotrypaea*. The two species, *N. harmandi* and *N. japonica*, were closely related with each other, while a Kimura-2-parameter (K2P) distance value for COI gene nucleotide sequence, which can be an index for species delimitation (e.g. Hebert et al. 2003), was 6.67% between these species. Given that the maximum intraspecific K2P value is shown to be no more than 5% in Decapoda (Costa et al. 2007; Matzen da Silva et al. 2011; Raupach et al. 2015), *N. harmandi* can be phylogenetically marginally separated as a species from *N. japonica*.

**Figure 1.**
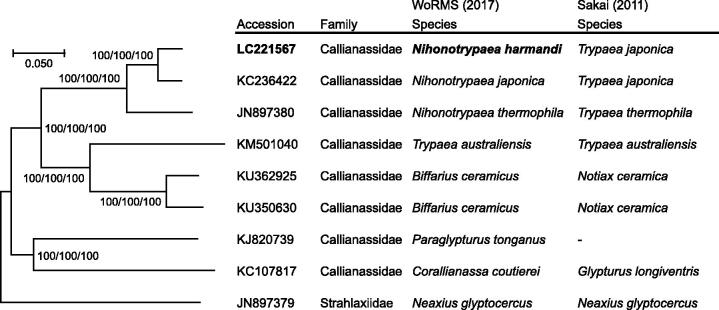
Phylogenetic relationships of the callianassid ghost shrimps inferred from a combined analysis of 13 CDSs and 2 rRNAs using NJ (K2P model), ML (GTR + I + G model) and MP methods each with 1,000 replicates of bootstrap. There were a total of 12,675 positions in the dataset. The tree shown is an NJ tree, and the ML and MP trees were the same topology. Numbers above nodes are bootstrap support values (NJ/ML/MP). *Neaxius glyptocercus* (accession number: JN897379) was used as outgroup. Classifications of species proposed by WoRMS Editorial Board (2017) and Sakai (2011) are listed. The original species name (source organism in GenBank) of the sequences used for tree reconstructions are listed as follows: KC236422 (*Nihonotrypaea japonica*), JN897380 (*Nihonotrypaea thermophile*), KM501040 (*Trypaea australiensis*), KU362925 (*Callianassa ceramica*), KU350630 (*Callianassa ceramica*), KJ820739 (*Paraglypturus tonganus*), KC107817 (*Corallianassa coutierei*), JN897379 (*Neaxius glyptocercus*).
